# Interleukin-35 as a key immunoregulatory mediator in steroid-hyporesponsive severe asthma

**DOI:** 10.3389/fimmu.2026.1790621

**Published:** 2026-03-06

**Authors:** Jehan Al-Matouq, Baraa Khalid Salah Al-Sheakly, Narjes Saheb Sharif-Askari, Rabih Halwani, Fatemeh Saheb Sharif-Askari

**Affiliations:** 1 Department of Pharmacy Practice and Pharmacotherapeutics, College of Pharmacy, University of Sharjah, Sharjah, United Arab Emirates; 2 Research Institute for Medical and Health Sciences, University of Sharjah, Sharjah, United Arab Emirates; 3 Department of Clinical Sciences, College of Medicine, University of Sharjah, Sharjah, United Arab Emirates; 4 Health and Wellbeing, NEOM, Tabuk, Saudi Arabia; 5 King Saud University Celiac Disease Research Chair, Department of Pediatric, College of Medicine, King Saud University, Riyadh, Saudi Arabia; 6 Prince Fahad Bin Sultan chair for Biomedical Research, University of Tabuk, Tabuk, Saudi Arabia; 7 College of Medicine, Alfaisal University, Riyadh, Saudi Arabia

**Keywords:** biomarker stratification, corticosteroid resistance, glucocorticoid receptor signaling, immune regulation, interleukin-35, MAPK, neutrophilic asthma, NF-κB

## Abstract

Severe asthma remains a major unmet clinical challenge, largely due to corticosteroid hyporesponsiveness in a subset of patients. Despite high-dose inhaled or systemic corticosteroids and targeted biologics, chronic airway inflammation often persists, particularly in T helper 2 (Th2)-low, neutrophilic, and mixed inflammatory phenotypes. Corticosteroid failure in severe asthma reflects not only excessive inflammation but a fundamental breakdown of immune regulatory mechanisms. At the molecular level, steroid hyporesponsiveness is associated with impaired glucocorticoid receptor (GR) signaling, including an altered GRα/GRβ balance, sustained activation of mitogen-activated protein kinase (MAPK) and nuclear factor kappa B (NF-κB) pathways, oxidative stress–mediated histone deacetylase 2 (HDAC2) dysfunction, and epigenetic stabilization of pro-inflammatory transcription. Concurrently, regulatory immune networks—particularly regulatory T and B cells that normally enforce immune tolerance and promote inflammatory resolution—are quantitatively and functionally compromised. Although biologics targeting immunoglobulin E (IgE), interleukin-5 (IL-5)/IL-5 receptor alpha (IL-5Rα), and IL-4 receptor alpha (IL-4Rα) have improved type-2-high asthma, their efficacy in steroid-hyporesponsive disease remains limited, as they do not restore immune regulation or glucocorticoid sensitivity. In this context, IL-35 has emerged as a uniquely positioned immunoregulatory cytokine. Produced mainly by regulatory T and B cells, IL-35 suppresses Th17-driven and innate immune inflammation, inhibits MAPK and NF-κB signaling, expands regulatory immune networks through infectious tolerance, and stabilizes epithelial barrier integrity. Importantly, IL-35 restores corticosteroid sensitivity in experimental models by targeting key drivers of steroid resistance. This review highlights IL-35 as a potential therapeutic target for managing steroid-hyporesponsive severe asthma by linking asthma endotypes, steroid resistance mechanisms, and IL-35 biology.

## 1 Introduction: steroid-hyporesponsive asthma as a failure of immune regulation

Asthma is a heterogeneous chronic inflammatory disease of the airways affecting more than 300 million individuals worldwide and remains a major cause of morbidity, healthcare utilization, and impaired quality of life. Although the majority of patients achieve adequate disease control with inhaled corticosteroids (ICS), either alone or in combination with long-acting bronchodilators, approximately 5–10% develop severe asthma that remains poorly controlled despite optimized therapy. This subgroup, commonly referred to as severe or difficult-to-treat asthma, accounts for a disproportionate share of asthma-related hospitalizations, exacerbations, healthcare costs, and mortality (1–3).

A defining characteristic of severe asthma is hyporesponsiveness to corticosteroids, manifested by persistent airway inflammation and ongoing symptoms despite high-dose inhaled or systemic glucocorticoid treatment. Importantly, increasing corticosteroid dosage rarely restores disease control and instead exposes patients to substantial systemic adverse effects, including metabolic dysfunction, osteoporosis, hypertension, and immunosuppression. Consequently, steroid-hyporesponsive asthma represents a major unmet clinical need and a critical limitation of current therapeutic strategies (4–7).

Historically, corticosteroid hyporesponsiveness has been attributed to excessive inflammatory burden or disease severity. However, accumulating experimental and clinical evidence challenges this interpretation and suggests that steroid failure reflects qualitative defects in immune regulation rather than inflammation alone. In severe asthma, inflammatory activity is often driven by pathways that are intrinsically less sensitive to glucocorticoid suppression, including neutrophilic inflammation, T helper 17 (Th17)-associated immune responses, epithelial alarmin signaling, and innate immune activation. These pathways persist despite aggressive corticosteroid therapy, indicating a fundamental breakdown in endogenous mechanisms that normally restrain immune activation (5, 8, 9).

At the molecular level, corticosteroid hyporesponsiveness is associated with impaired glucocorticoid receptor (GR) signaling, including reduced GR alpha (GRα) activity, increased expression of the dominant-negative GR beta (GRβ) isoform, defective induction of anti-inflammatory genes, and sustained activation of inflammatory kinase pathways (10–13). In parallel, regulatory immune networks—particularly regulatory T cells (Tregs) and regulatory B cells (Bregs), which normally enforce immune tolerance and resolution of inflammation—are quantitatively and functionally compromised in severe asthma. Together, these alterations create a permissive immunological environment in which inflammation persists despite corticosteroid exposure, rendering standard anti-inflammatory therapy ineffective (2, 14).

The clinical relevance of this regulatory failure is underscored by the limited efficacy of currently approved biologic therapies in steroid-hyporesponsive disease. While monoclonal antibodies targeting immunoglobulin (IgE), interleukin-5 (IL-5), IL-5 receptor alpha (IL-5Rα), and interleukin-4 receptor alpha (IL-4Rα) have transformed the management of eosinophilic, type-2-high asthma, a substantial proportion of patients—particularly those with Th2-low, neutrophilic, or mixed inflammatory phenotypes—derive limited benefit. These therapies effectively suppress selected inflammatory mediators but fail to correct the underlying dysregulation of glucocorticoid responsiveness and immune control that characterizes steroid-hyporesponsive asthma (15–17).

Collectively, these observations support a conceptual shift in our understanding of severe asthma. Rather than viewing steroid-hyporesponsive asthma as a condition of uncontrolled inflammation alone, it should be regarded as a disorder of immune regulatory failure, in which defective counter-regulatory mechanisms permit sustained inflammatory signaling despite corticosteroid exposure. This perspective carries important therapeutic implications, suggesting that effective intervention should reinforce endogenous immune regulation while restoring glucocorticoid responsiveness.

In this context, interleukin-35 (IL-35) has emerged as a compelling candidate for therapeutic exploration. IL-35 is a regulatory cytokine produced predominantly by Tregs and Bregs and is essential for maximal suppressive immune function. Unlike currently approved biologics that primarily target upstream inflammatory mediators, IL-35 exerts pleiotropic regulatory effects that integrate suppression of inflammatory signaling with reinforcement of endogenous immune control mechanisms. These properties position IL-35 as a mechanistically non-redundant mediator capable of addressing multiple pathogenic nodes underlying steroid hyporesponsiveness (18–20).

Despite growing interest in IL-35 biology across inflammatory diseases, its potential role in regulating corticosteroid responsiveness in asthma has not been systematically examined. No comprehensive review has integrated IL-35 signaling with the molecular mechanisms of steroid resistance, limitations of existing biologics, and emerging translational strategies in severe asthma. Addressing this gap is timely, given the increasing recognition of immune regulation as a therapeutic target and the persistent clinical challenge posed by steroid-hyporesponsive disease.

In this review, we synthesize current evidence defining the molecular and immunological basis of corticosteroid hyporesponsiveness in asthma, critically evaluate the limitations of existing therapeutic approaches, and examine IL-35 as a novel regulatory axis with the potential to restore steroid sensitivity. We further discuss biomarkers for patient stratification and emerging strategies for IL-35 delivery, positioning IL-35-based interventions as a promising avenue for precision therapy in severe asthma.

## 2 Asthma endotypes and inflammatory phenotypes underlying steroid hyporesponsiveness

Steroid hyporesponsiveness in asthma is closely linked to distinct inflammatory endotypes, particularly Th2-low phenotypes characterized by neutrophilic, mixed granulocytic, and innate immune–driven inflammation rather than classical type-2 eosinophilia (4–6). While Th2-high asthma—driven by IL-4, IL-5, IL-13 and epithelial alarmins—is generally corticosteroid sensitive, subsets show variable responses due to modifying factors such as obesity and epithelial injury (5, 8, 15, 21–23).

In contrast, Th2-low asthma is dominated by neutrophils and Th17- and Th1-associated cytokines (IL-17, tumor necrosis factor-α, interferon-γ). These pathways are intrinsically resistant to corticosteroid suppression and actively interfere with GR signaling through mitogen-activated protein kinase (MAPK) and nuclear factor kappa B (NF-κB) pathways (2, 5, 8, 12, 24). Epithelial alarmins, particularly IL-33, further amplify both type-2 and non–type-2 inflammation and correlate with poor steroid responsiveness (9, 22, 25, 26). Across these endotypes, defective regulatory T- and B-cell function emerges as a unifying feature that permits persistent inflammation despite therapy, providing a mechanistic framework for corticosteroid failure and a rationale for regulatory-focused interventions such as IL-35 (2, 5, 8, 27–29).

## 3 Molecular mechanisms underlying corticosteroid hyporesponsiveness in severe asthma

Corticosteroid hyporesponsiveness in severe asthma arises from convergent molecular defects that disrupt GR signaling despite adequate drug exposure. These defects include an imbalance favoring the dominant-negative GRβ isoform and aberrant post-translational modification of GRα, which together impair nuclear translocation and transcriptional activity (10, 12, 24, 30). As a consequence, defective GR transactivation leads to insufficient induction of key anti-inflammatory genes, including mitogen-activated protein kinase phosphatase-1/dual-specificity phosphatase 1 (MKP-1/DUSP1), permitting sustained activation of MAPK pathways (13, 31–33).

Persistent activation of MAPK family members—p38 MAPK, extracellular signal-regulated kinase (ERK), and c-Jun N-terminal kinase (JNK)—further interferes with GR function and reinforces steroid resistance. Excessive inflammatory kinase signaling and dominant NF-κB activity mutually antagonize GR-mediated transcriptional repression, promoting persistent cytokine production while further enhancing GRβ expression (12, 24, 34–36). In parallel, oxidative and nitrative stress inactivate histone deacetylase 2 (HDAC2), a critical cofactor required for glucocorticoid-dependent transrepression, resulting in sustained NF-κB–driven transcription despite steroid therapy (36–38). These signaling abnormalities are stabilized by epigenetic- and microRNA-mediated mechanisms, generating self-sustaining inflammatory loops—often linked to Th17 and innate immune pathways—that render conventional corticosteroids ineffective and underscore the need for therapies capable of restoring endogenous regulatory mechanisms (24, 37, 39).

## 4 Limitations of current biologic therapies in steroid-hyporesponsive asthma

Current biologic therapies have significantly improved clinical outcomes in selected patients with severe asthma by targeting type-2 inflammatory pathways, particularly IgE, IL-5/IL-5Rα, and IL-4Rα. These agents have reduced exacerbation rates and corticosteroid use in patients with Th2-high disease (7, 16, 39–43). However, their efficacy is limited in Th2-low, neutrophilic, or mixed phenotypes, in which patients frequently remain steroid dependent and poorly controlled (3, 5, 6).

Crucially, these biologics suppress downstream effector cytokines without addressing the core molecular drivers of corticosteroid hyporesponsiveness. These include impaired GRα signaling, increased GRβ expression, persistent activation of p38 MAPK and NF-κB pathways, oxidative stress–mediated HDAC2 dysfunction, and epigenetic dysregulation (8, 12, 15, 42). Moreover, current biologic therapies fail to restore deficient immune regulatory networks, particularly regulatory T- and B-cell function, thereby allowing Th17- and innate-driven inflammation to persist despite treatment (2, 6, 8, 44). Collectively, these limitations indicate that single-cytokine neutralization is insufficient to reverse steroid resistance and highlight the need for regulatory-focused therapies capable of restoring immune balance and corticosteroid responsiveness, exemplified by IL-35–based approaches (18, 20, 45).

## 5 Biology and regulatory signaling of interleukin-35

IL-35 is a member of the IL-12 cytokine family with distinctive structural, cellular, and functional properties that set it apart from other family members. In contrast to IL-12, IL-23, and IL-27—which primarily promote inflammatory T-cell differentiation—IL-35 functions as a potent immunoregulatory cytokine that is essential for immune tolerance and resolution of inflammation. Its biological profile is uniquely aligned with the regulatory defects observed in steroid-hyporesponsive asthma, positioning IL-35 as a compelling candidate for therapeutic exploration (46, 47).

### 5.1 Structural features and position within the IL-12 cytokine family

IL-35 is a heterodimeric cytokine composed of two subunits: Epstein–Barr virus-induced gene 3 (EBI3) and the p35 subunit encoded by IL-12A. Both subunits are shared with other members of the IL-12 cytokine family—EBI3 with IL-27 and p35 with IL-12—their unique pairing in IL-35 confers distinct biological properties and signaling functions (18, 46).

Functionally, IL-35 is the only IL-12 family cytokine whose dominant role is immune suppression rather than immune activation. Whereas IL-12 promotes Th1 differentiation, IL-23 stabilizes Th17 responses, and IL-27 exerts context-dependent immunomodulatory effects, IL-35 consistently suppresses effector T-cell proliferation and inflammatory cytokine production. This unique functional orientation reflects both its restricted cellular sources and its unconventional receptor signaling mechanism (46, 48, 49).

### 5.2 Cellular sources of IL-35

IL-35 is produced predominantly by regulatory lymphocyte populations. Tregs were the first identified source of IL-35, and IL-35 expression has been shown to be essential for maximal Treg suppressive function. Genetic deletion of either IL-35 subunit in Tregs markedly impairs their ability to suppress effector T-cell responses, demonstrating that IL-35 is not functionally redundant with other regulatory cytokines such as IL-10 or transforming growth factor beta (TGF-β) (18, 48).

Bregs represent a second major source of IL-35. IL-35-producing Bregs suppress Th1- and Th17-mediated immune responses and promote immune tolerance through coordinated secretion of IL-35 and IL-10. Importantly, B-cell-derived IL-35 appears to play a non-overlapping role in immune regulation, as selective loss of IL-35 expression in B cells results in exaggerated inflammatory responses despite intact Treg function (19, 44).

Beyond adaptive immune cells, IL-35 expression has also been reported in myeloid populations, including macrophages under inflammatory conditions. This broader cellular distribution suggests that IL-35 participates in multilayered regulation of immune responses, extending beyond classical lymphocyte-mediated tolerance and contributing to immune homeostasis in inflamed tissues (50–52).

### 5.3 IL-35 receptor usage and signaling pathways

A defining feature of IL-35 biology is its unconventional receptor usage. In T cells, IL-35 signals primarily through a heterodimeric receptor composed of IL-12 receptor β2 (IL-12Rβ2) and glycoprotein 130 (gp130). This receptor configuration differs from those utilized by IL-12 and IL-27 and enables unique downstream signaling events (46, 49).

Engagement of the IL-12Rβ2–gp130 receptor complex activates a signal transducer and activator of transcription 1 and 4 (STAT1:STAT4) heterodimer, a signaling signature that is unique to IL-35. The STAT1/STAT4 complex translocates to the nucleus and drives transcription of both IL-35 subunits, thereby establishing a feed-forward loop that sustains IL-35 production and regulatory activity. This self-reinforcing signaling mechanism contributes to the durability and stability of IL-35-mediated immune suppression (49).

In addition to heterodimeric receptor signaling, IL-35 can signal through homodimeric receptor combinations, including IL-12Rβ2–IL-12Rβ2 and gp130–gp130. However, these configurations are generally less efficient in mediating suppressive effects. Receptor expression is dynamically regulated by immune activation, with IL-12Rβ2 expression increasing following T-cell receptor stimulation and exposure to inflammatory cytokines, thereby modulating cellular responsiveness to IL-35 (49).

In B cells, IL-35 signals predominantly through a receptor composed of IL-12Rβ2 and IL-27 receptor α (IL-27Rα), leading to activation of STAT1 and STAT3 pathways. This signaling promotes differentiation and expansion of IL-35-producing Bregs, reinforcing immune regulation through both autocrine and paracrine mechanisms (19).

### 5.4 Induction of infectious tolerance and regulatory network expansion

One of the most distinctive functional attributes of IL-35 is its ability to induce infectious tolerance. IL-35 drives the generation of induced regulatory T cells (iTR35), a population of suppressive T cells that produce IL-35 but not IL-10 or TGF-β. These cells propagate regulatory signals to neighboring immune cells, thereby amplifying immune suppression beyond the original Treg population (48, 53).

In addition, IL-35 promotes the expansion of regulatory B cells, establishing a bidirectional regulatory network between Tregs and Bregs. This coordinated regulation is particularly relevant in chronic inflammatory diseases, where sustained immune suppression requires reinforcement across multiple immune compartments and cellular lineages (19, 27, 44).

### 5.5 Extracellular vesicle–mediated IL-35 signaling

In addition to classical soluble cytokine signaling, IL-35 can be delivered via extracellular vesicles (EVs). Regulatory T cells release EVs bearing IL-35 on their surface, enabling direct interaction with recipient immune cells. EV-associated IL-35 has been shown to induce regulatory phenotypes in target cells that do not express forkhead box P3 (FoxP3), thereby extending the reach of immune regulation beyond conventional regulatory cell populations (48, 53).

This EV-mediated mechanism enables the dissemination of immunoregulatory signals beyond the originating cell population and is particularly relevant within tissue microenvironments such as the lung, where localized delivery of regulatory signals is critical for maintaining immune homeostasis and limiting chronic inflammation (53, 54).

### 5.6 Functional implications for chronic inflammatory disease

The biological properties of IL-35—including its restricted production by regulatory cells, unconventional receptor signaling, induction of infectious tolerance, and reinforcement of regulatory networks—distinguish it from other immunomodulatory cytokines. Together, these features enable IL-35 to suppress multiple inflammatory pathways simultaneously while promoting durable and self-sustaining immune regulation (46, 48, 49, 53).

Importantly, IL-35 does not merely dampen inflammatory responses but actively reshapes immune tolerance and resolution. This distinction is particularly critical in the context of steroid-hyporesponsive asthma, where disease persistence reflects a failure of immune regulation rather than excessive inflammation alone (39, 46, 48).

### 5.7 Relevance of IL-35 biology to steroid-hyporesponsive asthma

Several core aspects of IL-35 biology align closely with the molecular and immunological defects that characterize steroid-hyporesponsive asthma. Reduced Treg and Breg function, Th17 dominance, persistent inflammatory kinase activation, and impaired glucocorticoid receptor signaling all reflect a breakdown of regulatory control. IL-35’s capacity to expand regulatory immune populations, suppress inflammatory signaling pathways, and reinforce regulatory feedback loops positions it as a uniquely suited mediator to address these interconnected defects (39, 45, 48, 49).

Although IL-35 has been investigated in multiple autoimmune and inflammatory disease contexts, its specific role in modulating corticosteroid responsiveness in asthma has not been systematically examined. Understanding IL-35 signaling and regulatory function therefore provides a critical foundation for evaluating its therapeutic potential in steroid-hyporesponsive asthma (39, 46).

## 6 Interleukin-35 as a restorative regulator of corticosteroid responsiveness

The defining feature of steroid-hyporesponsive asthma is the failure of corticosteroids to adequately suppress airway inflammation despite sufficient drug exposure. As outlined in previous sections, this failure arises from convergent defects in GR signaling, persistent activation of inflammatory kinase pathways, dominance of Th17-driven and innate immune inflammation, and impairment of immune regulatory networks. A critical question, therefore, is whether a single therapeutic axis can simultaneously target these interconnected pathological processes. Emerging evidence suggests that IL-35 fulfils this criterion and functions as a restorative regulator of corticosteroid responsiveness (5, 8, 12, 45).

### 6.1 IL-35 suppresses inflammatory kinase signaling that drives steroid resistance

Excessive activation of MAPKs, particularly p38 MAPK, represents a central mechanism underlying corticosteroid hyporesponsiveness. p38 MAPK interferes directly with GR function through phosphorylation-dependent mechanisms and indirectly by sustaining transcription of pro-inflammatory genes. IL-35 has been shown to suppress MAPK signaling across multiple inflammatory contexts, including immune and epithelial cell populations relevant to lung disease (12, 13, 29, 45).

Experimental studies demonstrate that IL-35 treatment reduces p38 MAPK phosphorylation and downstream inflammatory gene expression in activated monocytes and macrophages. Importantly, suppression of p38 MAPK activity by IL-35 restores sensitivity to glucocorticoids, thereby lowering the corticosteroid concentration required to achieve anti-inflammatory effects. This observation is particularly relevant in severe asthma, where elevated p38 MAPK activity is a defining molecular abnormality (12, 45).

In addition to p38 MAPK, IL-35 attenuates activation of ERK and JNK pathways, further limiting activator protein-1 (AP-1)–dependent inflammatory transcription. Through coordinated suppression of MAPK signaling, IL-35 targets a major upstream driver of steroid resistance that is not adequately corrected by existing biologic therapies (34, 35, 45, 55).

### 6.2 Inhibition of NF-κB–dominated inflammatory programs

Persistent activation of NF-κB is a hallmark of steroid-resistant inflammation and a major antagonist of effective glucocorticoid signaling. IL-35 suppresses NF-κB activation through multiple mechanisms, including inhibition of inhibitor of κB (IκB) degradation, reduction of NF-κB nuclear translocation, and suppression of NF-κB–dependent cytokine transcription (24, 51).

In models of lung and systemic inflammation, IL-35 treatment significantly reduces expression of NF-κB target genes such as IL-6, TNF-α, and IL-8, cytokines that are poorly suppressed by corticosteroids in severe asthma. By dampening NF-κB activity, IL-35 alleviates one of the principal molecular barriers to effective glucocorticoid action (29, 36).

Importantly, suppression of NF-κB by IL-35 also limits feed-forward induction of pro-inflammatory kinase signaling, thereby interrupting self-sustaining inflammatory loops that reinforce steroid resistance and perpetuate airway inflammation (51, 52).

### 6.3 Suppression of Th17-driven and neutrophilic inflammation

Th17-mediated inflammation is a major contributor to steroid-hyporesponsive asthma and is intrinsically resistant to corticosteroid suppression (8). IL-35 exerts potent inhibitory effects on Th17 cell differentiation, cytokine production, and effector function (20).

Experimental studies demonstrate that IL-35 suppresses IL-17A and IL-17F production, reduces STAT3 activation, and limits neutrophil recruitment to inflamed tissues (20, 56). In models of neutrophilic airway inflammation, IL-35 treatment reduces airway hyperresponsiveness, inflammatory cell infiltration, and tissue damage. These effects directly counteract inflammatory pathways that are inadequately targeted by currently available biologic therapies (20, 57).

By suppressing Th17-driven inflammation, IL-35 targets a central immune axis underlying corticosteroid resistance and restores susceptibility of airway inflammation to glucocorticoid- mediated control (20). Evidence supporting IL-35 efficacy across airway and systemic inflammatory models, including mechanisms relevant to steroid resistance, is summarized in **Table 1**.

**Table 1:** IL-35 in preclinical models of asthma and other inflammatory diseases.

Disease/model	IL-35 form & route	Main immunological effect	Relevance to steroid resistance/asthma	Ref.
OVA-induced allergic asthma (mouse)	Adenoviral IL-35 gene (intranasal)	↓ Th2/Th17 cytokines, ↓ eosinophils, AHR; ↑ FoxP3^+^ Tregs	Demonstrates airway-targeted IL-35 can remodel both Th2 and Th17 axes	(57)
OVA-induced asthma (mouse)	Recombinant IL-35 (systemic or local)	↓ IL-5, IL-13, IL-17; ↓ eosinophils/neutrophils; ↑ Tregs	Shows broad suppression of allergic and neutrophilic inflammation	(20, 57)
OVA/IL-35 -induced airway inflammation	IL-35 administration	↓ IL-33-induced GATA-3, IL-5, IL-13; ↓ eosinophils, mucus	Targets alarmin-high, steroid-poorly responsive disease	(63)
Asthmatic children (clinical observational)	Endogenous IL-35 (serum, sputum)	Lower IL-35 in acute asthma; levels increase during recovery, inverse to Th2 cytokines	Suggests IL-35 deficiency during exacerbation and potential biomarker role	(58)
Steroid-resistant asthma (patients’ monocytes)	Recombinant IL-35 (*in vitro*)	↓ p38 phosphorylation, ↑ MKP-1, ↑ GR–GRE binding, ↓ IL-6; lowers dexamethasone IC_50_	Direct mechanistic proof that IL-35 restores glucocorticoid sensitivity	(45)
Acute lung injury (LPS/ARDS models)	Recombinant IL-35 (intraperitoneal)	Inhibits TLR4/NF-κB; ↓ IL-6/TNF-α; ↑ Tregs; protects lung structure	Supports role in severe lung inflammation beyond asthma	(51)
Acute kidney injury (LPS)	Recombinant IL-35 (intraperitoneal)	↓ NF-κB activation, oxidative stress, apoptosis; improved renal function	Confirms organ-protective, systemic anti-inflammatory potential	(75)
Type 1 diabetes (NOD mouse)	Systemic IL-35	Prevents progression; ↓ effector T cells; ↑ Tregs	Shows durable immune reprogramming, supports safety/efficacy concept	(66)
Collagen-induced arthritis	IL-35 (systemic or via L. lactis)	↓ Th17, IL-17, TNF-α; ↑ Tregs and IL-10; improved joint pathology	Demonstrates mucosal IL-35 delivery and systemic immune modulation	(74)
IBD/Colitis, psoriasis	Recombinant IL-35	↓ pro-inflammatory cytokines; improved histology	Highlights multi-tissue efficacy	(67)

### 6.4 Expansion of regulatory T and B cell networks

A defining feature of IL-35 biology is its capacity to expand regulatory immune populations. IL-35 induces the generation of iTR35, which produce IL-35 themselves and propagate suppressive signals through infectious tolerance (48). These cells suppress effector T-cell proliferation independently of IL-10 and TGF-β, thereby providing a complementary and non-redundant regulatory mechanism (18, 48).

In addition, IL-35 promotes differentiation and expansion of Bregs that secrete IL-35 and IL-10, further reinforcing immune tolerance. This coordinated expansion of regulatory T and B cell networks directly addresses the regulatory immune deficiency observed in severe asthma (27, 44).

Importantly, restoration of regulatory immune cell function has downstream consequences for inflammatory signaling, including suppression of Th17 activity, reduction of neutrophil recruitment, and stabilization of epithelial barrier integrity. Collectively, these effects create a permissive immunological environment for effective glucocorticoid action (20).

### 6.5 Effects of IL-35 on airway structural cells

Beyond immune modulation, IL-35 exerts direct protective effects on airway structural cells. In bronchial epithelial cells, IL-35 reduces activation of p38 MAPK and NF-κB in response to inflammatory stimuli, thereby limiting cytokine release and epithelial injury. IL-35 has also been shown to inhibit inflammatory forms of programmed cell death, such as pyroptosis, which contribute to alarmin release and amplification of airway inflammation (29).

**Figure 1:**
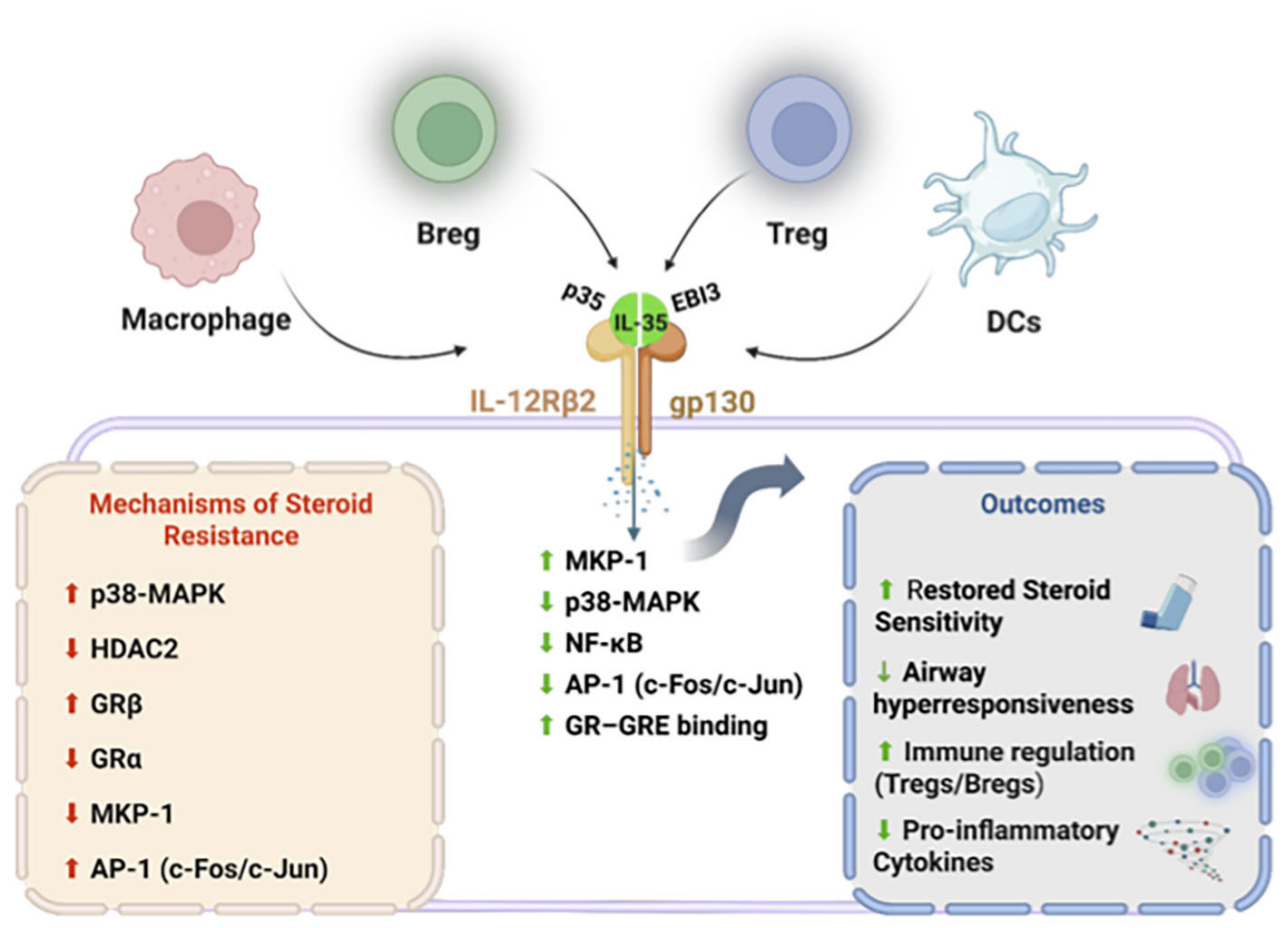
Interleukin-35 (IL-35), a heterodimeric cytokine composed of the p35 (IL-12A) and EBI3 (Epstein–Barr virus–induced gene 3) subunits and is produced by regulatory immune cells, including regulatory T cells (Tregs), regulatory B cells (Bregs), macrophages, and dendritic cells (DCs). IL-35 signals through the heterodimeric receptor complex IL-12Rβ2/gp130, leading to induction of mitogen-activated protein kinase phosphatase-1 (MKP-1) and suppression of p38 mitogen-activated protein kinase (p38-MAPK) and nuclear factor-kappa B (NF-κB) signaling. This results in reduced activator protein-1 (AP-1; c-Fos/c-Jun) activity and restoration of glucocorticoid receptor (GR) transcriptional function, as evidenced by enhanced GR binding to glucocorticoid response elements (GRE). In steroid-resistant conditions, these pathways are dysregulated and characterized by increased p38-MAPK activity, reduced histone deacetylase-2 (HDAC2) expression, increased GRβ, decreased GRα, and impaired MKP-1 signaling. Collectively, IL-35 signaling restores corticosteroid sensitivity, suppresses pro-inflammatory cytokine production, expands regulatory T-cell populations, and reduces airway hyperresponsiveness. Created with BioRender.com.

By stabilizing epithelial integrity and reducing alarmin-driven immune activation, IL-35 indirectly attenuates upstream triggers of steroid-resistant inflammation. This tissue-protective effect distinguishes IL-35 from biologic therapies that primarily target immune cells and highlights its potential to modulate the airway microenvironment. The integrated immunoregulatory and molecular mechanisms through which IL-35 restores corticosteroid responsiveness and attenuates airway hyperresponsiveness in severe asthma are summarized in **Figure 1**.

## 7 Biomarkers and patient stratification for IL-35–based therapy

The marked heterogeneity of severe asthma, together with the variable response to both corticosteroids and biologic therapies, underscores the need for biomarker-driven patient stratification strategies (3). Because IL-35 targets key regulatory and molecular defects that underlie corticosteroid hyporesponsiveness, identifying patients most likely to benefit from IL-35–based interventions will be essential for successful clinical translation. Emerging evidence suggests that IL-35 itself, in combination with markers of Th17 activity, neutrophilic inflammation, and glucocorticoid receptor dysfunction, may serve as informative biomarkers to guide precision therapy (45, 58).

### 7.1 IL-35 as a biomarker of immune regulatory competence

Both circulating and airway levels of IL-35 have been reported to be reduced in patients with severe and steroid-hyporesponsive asthma compared with individuals with steroid-sensitive disease and healthy controls. Reduced IL-35 levels correlate with increased disease severity, more frequent exacerbations, and impaired lung function, suggesting that IL-35 deficiency reflects a breakdown of endogenous immune regulation rather than simply an increased inflammatory burden (44, 45, 58).

Beyond absolute IL-35 concentrations, functional assessment of IL-35–producing regulatory populations may provide further insight. Reduced frequencies of IL-35–producing regulatory T cells (iTR35) and regulatory B cells has been associated with enhanced Th17 activity and coricosteroid resistance. Collectively, these observations support the concept that IL-35-related parameters may serve as biomarkers of immune regulatory competence in asthma (44, 45).

### 7.2 Th17-associated cytokines and neutrophilic inflammation

Given the central role of Th17-driven inflammation in corticosteroid hyporesponsiveness, biomarkers reflecting activation of this axis are likely to be particularly informative. Elevated levels of IL-17A, IL-17F, and IL-8 in sputum or bronchoalveolar lavage fluid correlate with airway neutrophilia, increased disease severity, and poor corticosteroid responsiveness. Similarly, increased neutrophil counts in sputum or peripheral blood identify patients with inflammatory phenotypes that are intrinsically less responsive to glucocorticoid therapy (5, 8, 59, 60).

Importantly, IL-35 suppresses Th17 differentiation and effector function, suggesting that patients with Th17-dominant inflammatory profiles may derive particular benefit from IL-35–based interventions. Monitoring changes in IL-17–associated biomarkers during IL-35-directed therapy could therefore provide early indicators of therapeutic response and inform treatment optimization (18, 20, 48, 59).

### 7.3 Alarmin-high phenotypes and epithelial injury

Epithelial-derived alarmins, particularly IL-33 and thymic stromal lymphopoietin (TSLP), play a central role in amplifying airway inflammation and contributing to corticosteroid hyporesponsiveness. Elevated levels of IL-33, soluble suppression of tumorigenicity 2 (sST2), and TSLP have been associated with severe asthma, airway remodeling, and frequent exacerbations (9, 26, 61).

Although alarmins are classically linked to type-2 inflammation, increasing evidence suggests that they also promote non–type-2 and mixed inflammatory responses, particularly in the context of epithelial injury and barrier dysfunction (9, 62). IL-35 has been shown to suppress alarmin-driven inflammatory signaling and protect epithelial integrity, suggesting that patients with alarmin-high disease may represent a distinct subgroup likely to benefit from regulatory-based therapeutic approaches (63, 64).

### 7.4 Composite biomarker profiles for precision therapy

Given the multifactorial nature of corticosteroid hyporesponsiveness, reliance on a single biomarker is unlikely to be sufficient for effective patient stratification. Instead, composite biomarker profiles that integrate regulatory, inflammatory, and molecular parameters are likely to provide greater predictive value for therapeutic response (39).

Such profiles may include reduced circulating or airway IL-35 levels, elevated Th17-associated cytokines, evidence of neutrophilic inflammation, high alarmin burden, and markers of impaired GR signaling. Integrating these parameters could enable identification of patients most likely to benefit from IL-35–based interventions while minimizing unnecessary exposure in non-responders (26, 45, 58).

### 7.5 Translational implications

Biomarker-guided patient stratification is particularly important for regulatory cytokine–based therapies, where broad immunosuppression must be avoided. By selectively targeting IL-35-based interventions to patients with demonstrable regulatory deficiency and corticosteroid resistance, it may be possible to maximize therapeutic efficacy while maintaining an acceptable safety profile (39).

Furthermore, longitudinal monitoring of IL-35 levels and downstream inflammatory and regulatory biomarkers during treatment may provide early indicators of therapeutic response and inform dose optimization. Such strategies align closely with emerging precision medicine frameworks in asthma and may accelerate clinical development and implementation of IL-35–based therapies.

## 8 Therapeutic delivery strategies and translational feasibility of IL-35

Although the immunoregulatory properties of IL-35 make it an attractive therapeutic candidate for steroid-hyporesponsive asthma, successful clinical translation depends critically on the development of safe, effective, and disease-appropriate delivery strategies. As a cytokine with potent immunomodulatory activity, IL-35 must be administered in a manner that maximizes therapeutic efficacy within the airway while minimizing systemic immunosuppression. Several delivery approaches have been explored in preclinical models, each associated with distinct advantages and limitations.

### 8.1 Recombinant IL-35 protein therapy

The most direct strategy for IL-35 delivery involves administration of recombinant IL-35 protein. In multiple preclinical models of inflammatory and autoimmune disease, systemic administration of recombinant IL-35 has been shown to suppress inflammatory cytokine production, inhibit effector T-cell responses, and expand regulatory immune populations (65–67). In models of airway inflammation, IL-35 protein therapy reduces inflammatory cell infiltration, airway hyperresponsiveness, and tissue damage (20, 57).

However, recombinant cytokine therapy presents significant challenges. Cytokines generally exhibit short circulating half-lives, necessitating frequent dosing to maintain therapeutic concentrations. Systemic exposure also raises concerns regarding off-target immunosuppression, increased susceptibility to infection, and interference with host defense mechanisms. These limitations have historically constrained the clinical use of regulatory cytokines and underscore the need for alternative delivery approaches tailored to chronic airway disease (68, 69).

### 8.2 Local and inhaled delivery approaches

For asthma, local delivery to the airway represents a particularly attractive strategy. Inhaled administration enables high local concentrations of therapeutic agents at the site of disease while minimizing systemic exposure. Preclinical studies employing intranasal delivery of IL-35 have demonstrated effective suppression of airway inflammation, attenuation of Th17-driven immune responses, and expansion of regulatory T-and B cell populations within the lung (20, 57, 70).

Despite these advantages, inhaled cytokine delivery poses technical challenges, including protein stability, aerosolization efficiency, and dosing precision. Nevertheless, advances in formulation science and inhalation technology have substantially improved the feasibility of delivering protein-based biologics via the respiratory route. These developments support the translational plausibility of inhaled IL-35 as a clinically viable approach.

### 8.3 Gene-based delivery strategies

Gene-based approaches offer the potential for sustained local expression of IL-35 within target tissues. Viral vectors encoding IL-35 subunits have been used in experimental models to induce prolonged IL-35 production, resulting in durable immunosuppressive effects (57, 71). In models of allergic airway inflammation, intranasal administration of IL-35–expressing vectors reduces airway hyperresponsiveness and inflammatory cell infiltration (57).

Despite these advantages, safety concerns remain a major barrier to clinical translation. Risks associated with viral vectors include immunogenicity, off-target effects, and challenges in controlling expression levels. In addition, regulatory hurdles and manufacturing complexity currently limit the near-term applicability of gene-based IL-35 delivery strategies for asthma.

### 8.4 Cell-based and extracellular vesicle–mediated approaches

Cell-based strategies, including adoptive transfer of IL-35–producing regulatory T cells or regulatory B cells, represent another potential delivery platform (18, 44). These approaches leverage endogenous regulatory networks and may enable highly targeted immune modulation. However, issue related to scalability, cost, and regulatory complexity present significant obstacles for widespread clinical application (72).

Extracellular vesicles (EVs) derived from IL-35–producing cells have emerged as an alternative means of delivering IL-35 in a biologically relevant context. EV-associated IL-35 can induce infectious tolerance and propagate regulatory signals to recipient cells (53). Although promising, EV-based therapies face challenges related to heterogeneity, manufacturing standardization, and biodistribution, which currently limit their current translational readiness.

### 8.5 Microbial and mucosal delivery platforms

Innovative delivery approaches using genetically engineered probiotic bacteria to produce IL-35 have demonstrated efficacy in models of intestinal and systemic inflammation (73, 74). These platforms exploit mucosal immune regulation and may offer advantages in terms of stability and cost-effectiveness. However, variability in microbial colonization, limited control over dosing, and interindividual differences in host-microbiome interactions remain significant challenges. Moreover, the applicability of these strategies to airway disease requires further investigation.

### 8.6 Translational considerations and safety

Regardless of delivery strategy, careful consideration of safety is essential. IL-35 exerts broad immunosuppressive effects, raising concerns regarding infection susceptibility and impaired immune surveillance, particularly with systemic administration (44, 66). Localized delivery to the airway may mitigate these risks by limiting systemic exposure while preserving pulmonary host defense mechanisms (57, 70).

Importantly, IL-35 does not induce global immunosuppression but rather reinforces regulatory pathways that are physiologically involved in maintaining immune homeostasis. This distinction may confer a more favorable safety profile compared with conventional immunosuppressive therapies, particularly when IL-35 is used in combination with existing corticosteroids rather than as monotherapy.

### 8.7 Toward clinical translation

Among the available strategies, inhaled IL-35 administration emerges as the most rational and clinically relevant approach for steroid-hyporesponsive asthma. By directly targeting the airway microenvironment, inhaled IL-35 has the potential to suppress steroid-resistant inflammatory signaling, restore glucocorticoid responsiveness, and reinforce immune regulation while minimizing systemic adverse effects (45, 57).

Future translational efforts should focus on optimizing IL-35 formulations for inhalation, defining dose–response relationships, validating biomarker-guided patient selection strategies, and evaluating efficacy in clinically relevant models of severe asthma. These steps will be essential for advancing IL-35 from experimental observation to therapeutic reality.

## 9 Final perspective and future directions

Steroid-hyporesponsive asthma remains one of the most challenging forms of chronic airway disease, characterized by persistent inflammation despite maximal corticosteroid therapy and increasingly recognized as a major unmet clinical need (3). The accumulating evidence reviewed here supports a paradigm shift in our understanding of this phenotype: corticosteroid failure in severe asthma reflects not merely excessive inflammatory burden, but a fundamental breakdown of immune regulatory mechanisms that normally restrain inflammatory signaling and restore tissue homeostasis (39). Although current biologic therapies have validated cytokine-targeted intervention as an effective strategy in selected patient populations-particularly those with type-2-high disease (15, 42), their limited efficacy in steroid-hyporesponsive asthma highlights a critical conceptual limitation. Suppression of individual inflammatory mediators is insufficient when GR signaling, inflammatory kinase control, and regulatory immune networks are concurrently compromised (5, 39). This realization highlights the need for therapeutic approaches that reprogram immune responses at a systems level rather than targeting isolated pathways.

Within this framework, IL-35 emerges from this framework as a mechanistically non-redundant regulatory cytokine uniquely positioned to address the core defects underlying steroid hyporesponsiveness. In contrast to existing biologics, IL-35 exerts coordinated effects across multiple pathogenic nodes, including suppression of MAPK and NF-κB signaling, restoration of GR function, inhibition of Th17-driven and neutrophilic inflammation, expansion of regulatory T and B cell populations, and protection of airway structural cells (20, 44, 51, 57). Through these integrated actions, IL-35 does not simply attenuate inflammation but actively restores the biological processes required for corticosteroid efficacy (45).

Importantly, IL-35-based strategies align closely with emerging precision medicine paradigms in asthma. Biomarker profiles reflecting regulatory deficiency, Th17 dominance, neutrophilic inflammation, and impaired GR signaling may enable identification of patients most likely to benefit from regulatory-focused interventions (5, 8, 45, 58). Moreover, localized delivery approaches, particularly inhaled IL-35, enhance translational feasibility by maximizing airway-specific effects while minimizing systemic immunosuppression and preserving host defense mechanisms (57, 70).

Looking forward, the therapeutic implications of IL-35 extends beyond asthma. By reinforcing endogenous immune regulation and restoring responsiveness to existing anti-inflammatory therapies, IL-35 represents a broader conceptual shift toward regulatory restoration as a treatment goal in chronic inflammatory diseases (65–67). Carefully designed preclinical studies and early-phase clinical trials integrating biomarker-guided patient selection will be essential to define the safety, efficacy, and optimal delivery strategies for IL-35-based interventions.

In conclusion, reframing steroid-hyporesponsive asthma as a disorder of immune regulatory failure provides a coherent framework for understanding therapeutic resistance and identifies IL-35 as a compelling candidate for next-generation treatment strategies. By targeting the regulatory deficit at the core of corticosteroid failure, IL-35 holds the potential to transform the management of severe asthma and redefine therapeutic approaches to treatment-refractory inflammatory disease.
